# Modelling societal preferences for automated vehicle behaviour with ethical goal functions

**DOI:** 10.3389/frai.2025.1676225

**Published:** 2025-12-10

**Authors:** Chloe Gros, Leon Kester, Marieke Martens, Peter Werkhoven

**Affiliations:** 1 Department Information and Computing Sciences, Universiteit Utrecht Faculteit Betawetenschappen, Utrecht, Netherlands; 2 Department Industrial Design, TNO, Utrecht, Netherlands; 3 Technische Universiteit Eindhoven, Eindhoven, Netherlands

**Keywords:** automated vehicles, ethical decision-making, ethical goal functions, discrete choice modelling, human-centered AI, AV ethics

## Abstract

**Introduction:**

As automated vehicles (AVs) assume increasing decision-making responsibilities, ensuring their alignment with societal values becomes essential. Existing ethical frameworks for AVs have primarily remained conceptual, lacking empirical operationalization. To address this gap, this study develops an Ethical Goal Function (EGF)—a quantitative model that encodes societal moral preferences for AV decision-making—within the theoretical framework of Augmented Utilitarianism (AU). AU integrates consequentialist, deontological, and virtue-ethical principles while remaining adaptable to evolving societal values. This work also proposes embedding the EGF into a Socio-Technological Feedback (SOTEF) Loop, enabling continuous refinement of AV decision systems through stakeholder input.

**Methods:**

The EGF was constructed using discrete choice experiments (DCEs) conducted with Dutch university students (N = 89). Participants evaluated AV-relevant moral scenarios characterized by six ethically salient attributes: physical harm, psychological harm, moral responsibility, fair innings, legality, and environmental harm. These attributes were derived from biomedical ethics and moral psychology and validated in prior AV ethics research. Using participants’ choices, a multinomial logit (MNL) model was estimated to derive attribute weights representing aggregate societal moral preferences. Model performance was evaluated using 5-fold cross-validation.

**Results:**

The MNL model produced stable attribute weights across folds, achieving an average predictive accuracy of 63.8% (SD = 3.3%). These results demonstrate that the selected attributes and underlying AU-based framework can meaningfully predict participants’ ethical preferences in AV decision scenarios. The EGF thus represents a data-driven, empirically grounded method for translating societal moral judgments into computationally usable parameters for AV decision-making systems.

**Discussion:**

This study contributes the first empirical operationalization of ethical frameworks for AVs through the development of an Ethical Goal Function and demonstrates how it can be embedded in a Socio-Technological Feedback (SOTEF) Loop for continuous societal alignment. The dual contribution advances both the theoretical grounding and practical implementation of human-centered ethics in automated decision-making. However, several limitations remain. The reliance on a Dutch university sample restricts cultural generalizability, and textual presentation may limit ecological validity. Future work should expand the cultural diversity of participants and compare alternative presentation modalities (e.g., visual, immersive) to better capture real-world decision contexts.

## 1 Introduction

Automated vehicles (AVs) are becoming increasingly integrated into modern transportation systems, offering the potential for significant improvements in efficiency, safety, and accessibility. However, as AVs begin to engage in more automated decision-making—particularly in situations involving ethical considerations—it becomes essential to define the principles needed to guide their behaviour explicitly. Unlike humans, AI systems, especially those based on deep learning, do not inherently reason using moral concepts or ethical reasoning (Gros et al., 2024). This makes it necessary to embed ethical decision-making structures into their operating frameworks.

To address this, several normative frameworks have been proposed to guide ethical behaviour in AVs. Notable among them are the Agent-Deed-Consequences (ADC) framework (Dubljević and Racine, 2014), which separates moral evaluation into three components, and Augmented Utilitarianism (AU) (Aliman and Kester, 2019a), which expands classical utilitarian ethics by integrating insights from fields such as moral psychology, neuroscience, and biomedical ethics, while dynamically adapting to evolving societal values and situational contexts. AU is particularly compelling because it operationalizes ethical reasoning through a structured set of well-defined, empirically grounded moral attributes (Gros et al., 2024; Gros et al., 2025b).

Building on the AU framework, this study aims to define an actual ethical goal function for AVs—a formal utility-based mechanism to evaluate and select actions in ethically complex scenarios according to predefined attributes. A goal or utility function allows an AV to assess the state of the world in terms of how well it aligns with mission-relevant objectives, including ethical imperatives (Aliman et al., 2019; Bradley et al., 1977). The utility function provides a numerical measure of effectiveness that the AV can use to compare alternative courses of action. By simulating potential outcomes and computing their utility, the AV can select the most ethically appropriate response, adapting in real-time as new information becomes available. This decision-making process is not a spontaneous or arbitrary choice made at the moment but is firmly aligned with a set of predefined ethical values and priorities embedded in the model.

A core challenge in this approach lies in determining the relative importance—or weight—of each ethical attribute within the function. In real-world traffic situations, decisions often require balancing competing ethical concerns such as minimising harm, respecting legality, and ensuring fairness. This study addresses that challenge through an empirical choice-model experiment designed to elicit public preferences and derive the appropriate attribute weights for an AV’s ethical goal function.

While several studies have discussed conceptual or normative frameworks for ethical AV decision-making—such as utilitarian models, deontological rules, or hybrid approaches (e.g., the Moral Machine experiment (Awad et al., 2018), the ADC framework (Dubljević and Racine, 2014), and Augmented Utilitarianism (Aliman and Kester, 2019a))—few have demonstrated how these theoretical principles can be operationalized into a computationally tractable model grounded in empirical human data. Most prior work stops at identifying relevant ethical dimensions without quantifying their relative weights or testing their predictive validity. This study explicitly addresses this gap by constructing and empirically estimating an Ethical Goal Function (EGF) through Discrete Choice Modelling, translating moral preferences into a formal decision-making structure. Furthermore, we extend existing literature by embedding the EGF within the proposed Socio-Technological Feedback (SOTEF) Loop, introducing a mechanism for continuous societal alignment. This dual contribution—(1) the empirical operationalization of ethical attributes into a predictive model and (2) its integration into an adaptive feedback framework—marks a novel and practical step toward making human-centered ethical reasoning computationally implementable in AV systems.

## 2 Methodology

### 2.1 Experimental goals

The objective of this paper is to demonstrate that it is possible to elicit an ethical goal function for AV decision-making based on experimental studies with human participants, acting as moral representatives of society. To achieve this, we aim to:

1. Elicit attribute weights for utility: Using a choice experiment, we will determine the weights assigned to different ethical attributes when participants are presented with specific AV decision-making scenarios. This will allow us to understand how individuals prioritise different moral and practical considerations in AV ethics for the scenarios tested.
2. Fit a model to the data to show the actual feasibility of an EGF based on real data: The collected choice data per scenario will be analysed using a multinomial logit model, enabling us to derive a decision-making framework that reflects best participant preferences across scenarios. This model will serve as the basis for a predefined ethical goal function that can be integrated into AV decision systems, ensuring ethical considerations are systematically incorporated into AV behaviour.

By fulfilling these goals, this study contributes to the ongoing effort to operationalize ethical decision-making for AVs, moving beyond theoretical frameworks to empirically grounded models that can inform real-world implementation.

### 2.2 Discrete choice modelling

In order to fulfil these goals, we are using a Discrete Choice Modelling approach. Discrete Choice Modelling is a method that derives preferences by observing participants’ preferred world states and fitting a model to that data (Koppelman et al., 2006). The process involves three main steps:

#### 2.2.1 Step 1: choosing the model type

The experimenter selects the type of choice model, including the number of attributes, attribute values, and the utility function.

#### 2.2.2 Step 2: gathering data

Data is gathered through controlled experiments, such as forced-choice experiments, where participants make decisions among hypothetical choices. These experiments present participants with sets of world states, and their choices reveal their implicit preferences. The number of questions in the experiment must balance data sufficiency and participant fatigue.

#### 2.2.3 Step 3: fitting the model

The final step is to fit the model to the dataset. The method for fitting depends on the chosen model type. This is done using statistical methods, such as maximum likelihood estimation, that infer the most probable parameter values given the data. The goal is to identify the combination of weights or coefficients that maximises the model’s predictive accuracy and reflects consistent decision patterns. This process transforms the experimental data into a working ethical goal function that can be applied and tested. Full implementation details, including the chosen estimation method and software tools, are provided in the technical appendix to support future replication and validation.

The advantage of this approach is that it infers ethical preferences from participants’ actual choices in forced-choice scenarios, rather than relying solely on direct self-reports or ratings of attribute importance. This can be more accurate, as people often struggle to articulate their true preferences. Additionally, this method is relatively cost-effective and straightforward to implement, making it suitable for iterative exploration of different attributes and models.

However, there are some disadvantages. The observed preferences are based on hypothetical scenarios, which may not perfectly align with real-world decisions. This assumption can be problematic in contexts where real-world pressures significantly influence decision-making. Furthermore, the model might capture simplified heuristics rather than true preferences, especially if the choices are overly complex. This issue is not unique to Discrete Choice Modelling and affects other preference elicitation methods as well. Therefore, after developing the EGF it is crucial to validate the model against real-world behaviour or realistic simulations to assess whether it accurately reflects actual decision-making and performs as intended in practice.

### 2.3 Model selection

To analyse the data collected from the choice experiment, we selected a multinomial logit (MNL) model. The MNL model is a widely used discrete choice model that estimates the probability of selecting a particular alternative based on the attributes associated with each option (Aggarwal, 2019; Chorus, 2015). It assumes that decision-makers evaluate alternatives independently and choose the option with the highest perceived utility.

The MNL model provides a straightforward way to estimate attribute weights, making it an efficient choice for studies where alternatives are relatively independent. Its structure allows for a clear interpretation of attribute effects on decision-making. Moreover, the MNL model is suitable for a limited number of attributes and levels, offering sufficient flexibility without unnecessary complexity. While more advanced models like the Mixed Logit model can account for preference heterogeneity and relax some assumptions of the MNL, they also require larger sample sizes and greater computational resources. For this proof-of-concept stage, where the primary goal was to establish a baseline ethical goal function and interpret general patterns in decision-making, the MNL model struck an appropriate balance between model complexity, interpretability, and practical feasibility. Future work could explore more sophisticated models like Mixed Logit to capture individual-level variation once larger datasets become available.

### 2.4 Data gathering

#### 2.4.1 Forced choice experiment

To determine the relative weights of the ethical attributes in AV decision-making, we designed a forced-choice experiment. In this type of experiment, participants are presented with two possible outcomes of an AV’s decision in an unavoidable dilemma and must choose the one they find more appropriate. Each scenario varies across predefined attributes, such as physical harm, moral responsibility, and legality, allowing us to observe trade-offs in decision-making. This approach ensures that participants actively engage with ethical dilemmas rather than rating attributes in isolation, enabling a more accurate estimation of their implicit preferences.

#### 2.4.2 Experimental design

##### 2.4.2.1 Attribute selection

As previously mentioned, this study is grounded in the framework of Augmented Utilitarianism (AU), a non-normative ethical model that integrates elements of virtue ethics, deontology, and consequentialism, as well as moral psychology and neuroscience (Aliman and Kester, 2019b, 2022). Unlike traditional utilitarian approaches that focus solely on maximizing overall happiness, AU introduces dynamic ethical goal functions that adapt to societal values and situational contexts. These goal functions quantify moral attributes and adjust decision-making processes, ensuring that AVs align with predefined ethical considerations.

Following this method, an initial set of ethical attributes was selected using the methodology proposed by Gros et al. (2025b), which adapts the principles of AU for application in real-world decision-making systems. Following this foundational selection, the attribute set was refined and extended through validation experiments. These studies involved both qualitative and quantitative assessments, such as attribute ratings and confidence measurements, to test the clarity, relevance, and ethical salience of each attribute across diverse scenarios (Gros et al., 2025b). Participant feedback and experimental data were used to remove ambiguous attributes, improve definitions, and incorporate new ones where ethical blind spots were identified.

Importantly, previous experiments focused on assessing the perceived relevance of individual attributes in isolation, i.e., how important a specific attribute is in a given type of situation. The goal there was to map the ethical landscape by identifying which dimensions participants consciously recognise as morally important. In contrast, in the current study, participants are asked to make trade-offs between attributes within a concrete scenario, requiring them to express not just whether an attribute matters, but how much it matters relative to others. This approach captures implicit ethical priorities and allows for the construction of a quantifiable ethical goal function, offering a more integrative and decision-oriented view of moral reasoning.

The final attribute set in Table 1 was selected through a rigorous process grounded in biomedical ethics, which focuses on minimising harm and prioritising human safety, principles closely aligned with ethical challenges in AV decision-making (Beauchamp and Childress, 2019). Biomedical ethics provides a practical framework for life-or-death dilemmas, making it suitable for mapping similar trade-offs faced by AVs, such as choosing between risking harm to different road users.

**Table 1:** Classification of attributes based on biomedical ethics and moral psychology.

Level 1	Level 2	Level 3	Level 4	Level 5
Patient	Utility	Harm	Physical Harm	Severity of damage
				Type of damage (temporary/permanent)
			Psychological Harm	Family Status
			Perceived Vulnerability	Age
				Physical condition
				Gender
			Environmental Harm	Nature damage
				Animal harm
				Property damage
		Social Utility	Social Status	Age
				Physical Condition
				Profession
				Gender
				Family status
	Fairness	Liability	Moral responsibility	/
		Fair opportunity	Fair innings	Age
Action of AV	Fairness	Liability	Legality	/
		Fair opportunity	Lottery	/
Actor (car)	Cost	Timeliness	Time of Arrival	/
		Financial Cost	Self-preservation	/
		Environmental Cost	Energy Efficiency	/

We structured the attributes into levels (see Table 1): Level 1 categorises the entity concerned (patient, action, or agent), based on dyadic morality theory (Schein and Gray, 2018). Level 2 reflects key ethical dimensions adapted from biomedical ethics: Utility, Cost, and Fairness.

- Utility captures the harm or benefit to individuals, including physical and psychological damage, as well as social utility (e.g., prioritising certain societal roles).
- Cost covers trade-offs like timeliness and vehicle damage, balancing safety against practical goals.
- Fairness involves moral responsibility (liability for risky behaviour), legality (compliance with laws), and fair opportunity principles such as the fair innings rule or lotteries in close cases.

The attribute framework used in this study is structured hierarchically, drawing on both moral psychology and biomedical ethics. At the foundational level, harm is defined according to dyadic morality theory, which emphasizes the roles of agent, action, and patient (Schein and Gray, 2018). Higher levels build on Beauchamp and Childress’ biomedical ethics principles—Utility, Justice, and Fairness—gradually refined into more specific and scenario-relevant attributes (Beauchamp and Childress, 2019).

This experiment focuses on Level 4 attributes, as they best capture morally salient distinctions in AV decision-making. These include:

- Physical Harm: Extent of bodily injury to an individual or group.
- Perceived Vulnerability: Non-physical vulnerability (e.g., disability) affecting perceived harm.
- Psychological Harm: Mental or emotional trauma to victims or close others.
- Social Utility: Perceived societal value of an individual (e.g., role, profession).
- Moral Responsibility: Degree to which the individual caused the risky situation.
- Legality: Whether the individual’s behaviour violated traffic laws.
- Fair Innings: Whether the individual had the chance to live a “full” life.
- Lottery: Random selection as an ethical decision rule.
- Time Delay: Impact on timely arrival due to the AV’s decision.
- Car Preservation: Damage to the vehicle and its consequences for the owner.

For this study, we selected the five attributes that were consistently ranked as most relevant in previous experiments, and added Environmental Harm, a new attribute introduced based on participant suggestions that had not been tested previously (Gros et al., 2025b). Each attribute was divided into four distinct levels. For instance, the attribute “Physical Harm” ranges from “No harm” to “Fatality,” including intermediate levels such as “Minor injury” and “Severe injury.” For a comprehensive overview of the attribute levels used in this experiment, refer to Table 2.

**Table 2:** Detail of attributes and levels.

Attribute	Level 1	Level 2	Level 3	Level 4
Physical Harm	No harm	Minor injury (e.g., small fracture)	Severe injury (e.g., disability)	Fatality
Psychological damage	No Distress (e.g., the individual remains calm and unaffected)	Low Distress (e.g., momentary fear or nervousness)	Moderate Distress (Significant emotional impact, short-term trauma, e.g., reluctance to use roads again temporarily)	Severe Trauma (long-lasting psychological harm, e.g., post-traumatic stress disorder (PTSD), depression)
Moral responsibility	Fully responsible (e.g., running onto the road without looking)	Partially responsible (distraction)	No responsibility (on crosswalk)	Victim of external factors (pushed)
Fair innings	Fully lived life (80 + years)	Close to full life (50–70 years)	Partial life (20–49 years)	Minimal life lived (child)
Legality of the action	Fully legal (following all rules)	Minor infraction (e.g., briefly crossing into a bus-only lane to avoid an unexpected obstacle)	Moderate infraction (e.g., speeding by 10–20 km/h in a residential area to swerve around an unexpected obstacle)	Severe violation (e.g., running a red light or driving on the wrong side of the road to avoid an unexpected obstacle)
Environmental harm	Minimal environmental impact	Low damage (e.g., minor pollution, minimal scratches or disturbance to roads or vegetation)	Moderate damage (e.g., significant emissions, cracked roads or toppled light poles)	High impact (e.g., major pollution, large-scale destruction of surrounding environment and infrastructures)

##### 2.4.2.2 Questionnaire structure

The experiment was conducted through an online questionnaire consisting of the following sections:

- Introduction: Participants were first introduced to the objectives of the study and provided with clear definitions for each of the six attributes used in the scenarios.
- Tutorial: A short tutorial explaining the decision task using a simplified example. Participants were guided through the structure of a sample choice set to ensure they understood how to interpret and compare scenarios.
- Main Experiment: Each participant was presented with 24 choice scenarios. Every scenario required them to choose between two AV actions (with different attribute profiles) and a status quo option. Scenarios were randomized in order to reduce ordering effects. At least one dominance test and one repeated choice set were included to assess consistency.
- Follow-Up Questions: After the main choice tasks, participants were asked follow-up questions to help assess sensitivity to the attribute trade-offs and identify gaps in the attribute set:
  - “How confident did you feel about your decisions?” (5-point Likert scale)
  - “Do you think these attributes completely define AV decision-making? If not, what should be added?”
  - “Which attributes should always or never be taken into account in AV decision-making?”
- Demographic Section: Finally, participants answered demographic and contextual questions, including:
  - Age, gender, education level, and employment status
  - Familiarity with AVs
  - Primary mode of transportation

##### 2.4.2.3 Choice sets design

To design a forced choice experiment, we needed to create choice sets comprising of two scenarios differing based on their attributes. In line with the Augmented Utilitarianism (AU) framework and the design approach used in previous studies (Gros et al., 2025b), we decided to divide the scenarios into two categories: critical and non-critical. Critical scenarios involve a high probability of harm, whereas non-critical scenarios involve either no harm or only minor injuries. This distinction was validated by the results of previous studies showing significant differences in the ratings of several attributes for critical and non-critical scenarios, namely Physical Harm, Psychological Harm, and Legality of the Action of the AV.

In practice, this means that each choice task presented to participants included options with either non-severe outcomes (Physical Harm levels 1—no harm, or 2—minor injury) or severe outcomes (Physical Harm levels 3—serious injury, or 4—fatality), but not a mix of both within the same task. Including both severe and non-severe harm options in a single task would likely render other moral attributes comparatively irrelevant, as participants would consistently favour avoiding physical harm regardless of other factors. By separating the harm levels, the study allowed for a more meaningful analysis of how participants weigh non-physical ethical considerations within comparable risk contexts.

Further, feedback from the pilot study indicated that participants were confused by scenarios in which the severity of physical and psychological harm did not align, particularly in critical cases. Several participants noted that presenting severe or fatal physical harm alongside low or no psychological harm felt unrealistic or incoherent, undermining the plausibility of the scenario. To address this, the Psychological Harm attribute was aligned with critical scenarios by setting it to levels 3—moderate distress, or 4—severe trauma. All other attributes remained unchanged.

To construct the choice sets for the experiment, we used the Ngene software to generate an efficient experimental design. Unlike orthogonal designs—which ensure statistical independence between attribute levels but can be inefficient in estimating model parameters—Ngene uses D-efficient design algorithms that minimise the determinant of the covariance matrix of the parameter estimates. This approach optimises the statistical information gained from each choice task, allowing for more precise and reliable estimation of attribute weights with fewer scenarios (ChoiceMetrics, 2024). By incorporating prior information and expected effect directions, this design ensures that the experiment is tailored to the model’s structure and goals, resulting in better model performance and reduced respondent fatigue.

**Figure 1:**
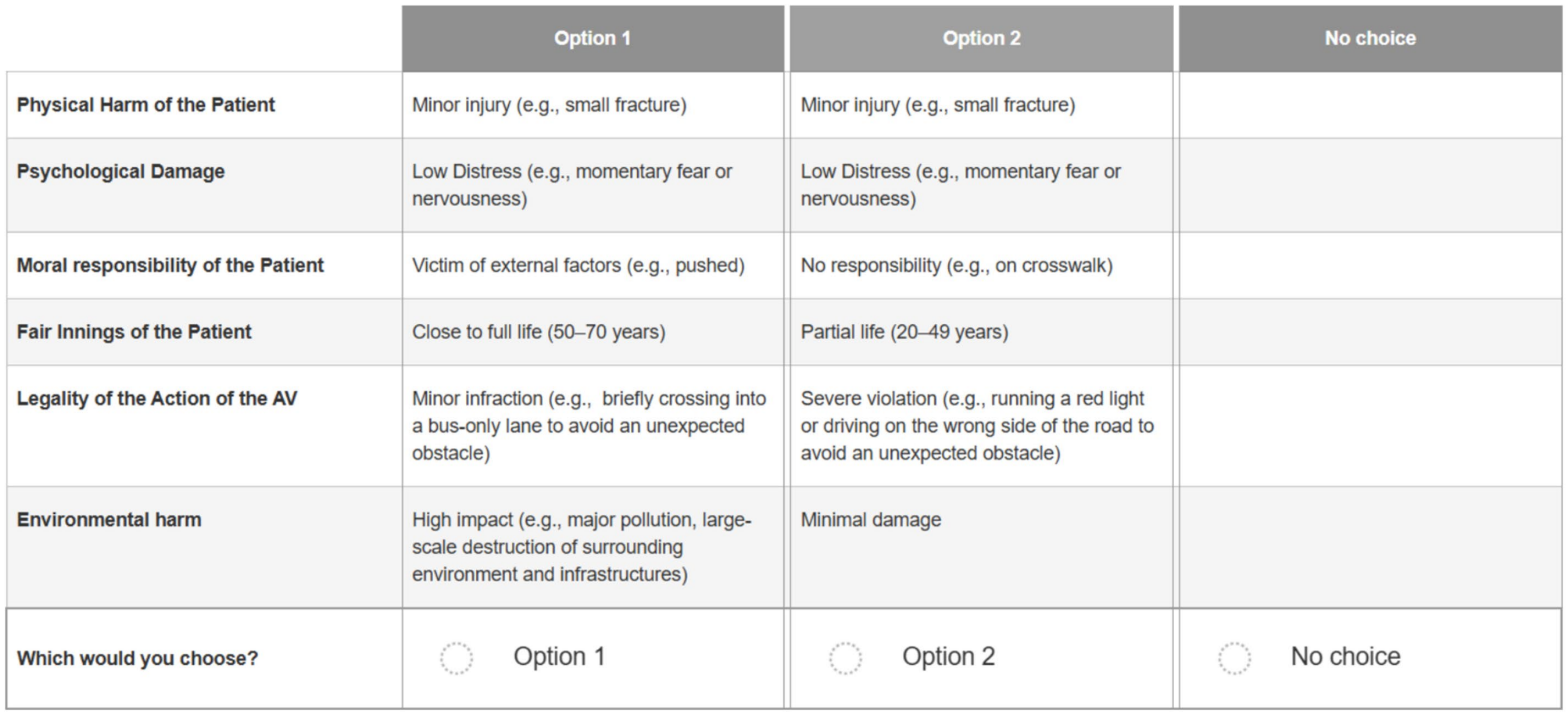
Example of scenario as seen by the participants.

A set of 30 scenarios was randomly generated using Ngene, including 12 critical scenarios and 18 non-critical scenarios. The difference in the number of critical and non-critical scenarios resulted from the random generation process. Participants were presented with a series of 24 choice sets, randomly selected from the set, each requiring them to evaluate hypothetical scenarios involving two AV actions and a status quo option (i.e., the AV takes no action), based on the previously detailed attributes (see Figure 1).

An important methodological consideration concerns the format in which scenarios are presented to participants. Prior research has demonstrated that different presentation methods (e.g., textual descriptions, schematic drawings, or dynamic 3D animations) can lead to different moral responses, with more immersive formats often eliciting stronger utilitarian tendencies (Gros et al., 2025a). In this study, however, the choice for textual descriptions was motivated by the complexity of modelling scenarios involving six attributes simultaneously. Textual presentation offered the clearest way to explicitly combine and communicate these attributes while minimising perceptual confounds. Although this format was well suited for our initial proof-of-concept, we acknowledge that future studies could benefit from systematically comparing textual, schematic, and dynamic formats to better understand how scenario representation shapes societal preferences.

##### 2.4.2.4 Consistency checks

To ensure the quality and reliability of the data collected, several measures were implemented in the experimental design. First, the order of the choice sets was randomised to minimize ordering effects and reduce response biases. Second, a dominance test was included, featuring a choice set in which one option was clearly superior across all attributes. This helped identify inattentive or non-engaged participants (Abbey and Meloy, 2017). Finally, an explicit attention check was inserted into the survey to verify that respondents were carefully reading and following the instructions.

##### 2.4.2.5 Scenario realism and comprehension

To ensure that the text-based scenarios were both realistic and easy to interpret, several steps were taken. First, each scenario was pre-tested in a pilot study (N = 10), where participants were asked to comment on clarity, coherence, and perceived realism. Feedback from this pilot led to wording adjustments (e.g., simplifying phrasing and ensuring internal consistency between physical and psychological harm). Second, the final questionnaire included a tutorial with an annotated example explaining how to read and compare scenario descriptions, ensuring participants understood the structure before starting the main task. Third, we included follow-up questions asking participants to rate how clear and realistic they found the scenarios. The majority reported finding them understandable and plausible. Collectively, these steps ensure that the text-based presentation maintained both realism and cognitive accessibility.

#### 2.4.3 Participants

Participants were recruited on university campus through posters and announcements. A total of 89 individuals participated in the study. Following dominance and attention checks, the data of two participants was removed. The sample was predominantly composed of students (78%), reflecting the target demographic for this experiment, as we aimed to maintain a relatively homogeneous group in terms of age, educational background, and digital literacy. This approach is consistent with prior work in the field (Gros et al., 2025b). Most participants were between 18 and 24 years old (80%), with smaller groups aged 25–34 (18%) and 35–54 (2%).

In terms of gender, the sample included 47 males (53%), 41 females (46%), and 1 non-binary respondent (1%). Educational backgrounds varied: 34% had completed high school, 17% had some college education, 36% held a bachelor’s degree, and 13% had completed postgraduate degrees.

Regarding employment, 10% of participants were employed full-time (PhD students), 12% part-time, and 78% were students. Familiarity with automated vehicles was moderate, with 15% reporting being very familiar, 67% somewhat familiar, and 18% not familiar with AVs.

Primary modes of transportation varied, with 37% using public transport, 36% cycling, 19% walking, and 8% relying on personal cars. A small number used motorcycles or scooters (1%), and no participants selected ride-sharing services as their main mode.

### 2.5 Evaluation of the choice model

To evaluate the performance and reliability of the final choice model, we used a combination of statistical and behavioural criteria, including model fit and participant confidence. The choice models were constructed using the Biogeme software and estimated via its logit implementation, resulting in multinomial logit models. Model fit was primarily assessed using McFadden’s Rho-square (𝜌^2^), which approximates how well the model explains the observed choice behaviour. A higher Rho-square indicates a stronger explanatory power relative to a null model.

In addition to overall fit, we closely examined the utility weights for each attribute. Each coefficient was tested for statistical significance, confirming whether the attribute had a consistent and measurable impact on decisions. Furthermore, we analysed correlations between weights to detect any redundancy or multicollinearity between variables, ensuring that the model captures distinct and meaningful dimensions of ethical evaluation.

We also assessed sensitivity—whether participants adjusted their choices in response to changing attribute levels. This was confirmed through the model’s ability to detect predictable shifts in choice behaviour in scenarios where ethically salient attributes (e.g., physical harm, moral responsibility) varied. Such sensitivity is essential for demonstrating that participants were engaged and understood the ethical trade-offs presented.

Lastly, participant confidence was measured to evaluate the clarity and difficulty of the decision tasks. After selecting scenarios, participants were asked to rate how confident they felt in their decisions using a Likert scale. The resulting data provided insight into the perceived complexity of the scenarios and supported the overall reliability of the responses. High levels of reported confidence, paired with consistent and sensitive choices, suggest that participants understood the dilemmas and engaged meaningfully with the experiment.

## 3 Results

### 3.1 Choice model

#### 3.1.1 Modelling approach

Incorporating separate functions for critical and non-critical scenarios could offer a way to better capture the nuances of ethical decision-making. Critical scenarios, such as those involving life-or-death situations, may require different ethical considerations compared to non-critical ones, such as minor inconveniences or damages. However, even with these distinct functions, it is important that they remain part of a unified ethical goal function. This overarching goal function should reflect the vehicle’s broader ethical framework, ensuring that the AV’s decision-making process is coherent and consistent across varying levels of severity. Mathematically, this could be achieved by introducing weighted sums or multi-objective optimization techniques, where different ethical attributes (e.g., safety, fairness, efficiency) are balanced depending on the situation. The challenge lies in determining how to appropriately weight and integrate these multiple functions, particularly in ensuring that the AV does not prioritize one aspect of ethics (such as safety) to the detriment of others (such as fairness or transparency).

A two-stage decision model was implemented to reflect how participants approached ethical AV dilemmas involving varying degrees of physical harm. This structure was designed to mirror observed behaviour from the pilot study, in which physical harm consistently dominated other attributes in decision-making.

##### 3.1.1.1 Rule-based stage

In the first stage, the model applies a deterministic rule: if one scenario presented a lower level of physical harm than the other (using two harm groups: low harm = levels 0–1, high harm = levels 2–3), that option was selected as the preferrable option. This reflects the intuitive tendency of participants to prioritise avoiding severe physical harm above all other considerations.

##### 3.1.1.2 Utility-based stage

When both scenarios fell within the same harm group (e.g., both involved minor or both involved serious harm), the model transitions to a utility-based evaluation. A discrete choice model is applied to evaluate the remaining ethical dimensions. Two multinomial logit models are used to estimate utility weights of the six attributes: one for critical scenarios and one for non-critical scenarios.

Utility functions were defined for all three alternatives presented in each choice set:

- Options A and B: Modelled as linear combinations of the attributes.
- No Choice: Assigned a constant utility (ASC_NC) to capture any baseline tendency to reject both presented scenarios.

The two-stage decision model effectively integrates separate functions for critical and non-critical scenarios into a single ethical goal function for the AV. The overall ethical goal function G can be represented as:


$$G=\{\begin{array}{c} \text{if}\;H_{A}<H_{B},A\\ \text{if}\;H_{B}<H_{A},B\\ \text{if}\;H_{A}=H_{B},\{\begin{array}{c} \text{if}\;H_{A}=\mathrm{NC},\max(U_{\mathrm{NC}}(A),U_{\mathrm{NC}}(B))\;\\ \text{if}\;H_{A}=C,\max(U_{C}(A),U_{C}(B)) \end{array}\;\; \end{array}$$

With A and B the two options, H_X_ the harm group of option X (C: critical, NC: non-critical), U_NC_(X) the utility of scenario X using the non-critical goal function, and U_C_(X) the utility of scenario X using the critical goal function.

Model estimation was conducted using Pandas Biogeme 3.2.13, a Python-based package tailored for discrete choice modelling. The final model’s predictive performance was evaluated through choice prediction agreement and confusion matrices, providing insight into its ability to replicate actual participant decisions.

##### 3.1.1.3 Model estimation for critical scenarios

The multinomial logit model for critical scenarios was estimated using 837 valid choice observations, derived from 87 participants each completing a set of 24 scenarios, and included 23 parameters, comprising alternative-specific constants and utility weights for six scenario attributes. The model achieved a final log-likelihood of −523.92, corresponding to a rho-squared value of 0.4302, indicating a strong model fit and substantial explanatory power relative to the null model. Model selection criteria further supported the robustness of the specification, with an Akaike Information Criterion (AIC) of 1093.85 and a Bayesian Information Criterion (BIC) of 1202.63. The alternative-specific constants showed strong preferences for selecting either action scenario over the opt-out option: ASC_A (β = 2.44) and ASC_B (β = 2.15) were both highly significant (*p* < 0.001), while the no-choice alternative was strongly disfavoured (ASC_NC = −4.60, *p* < 0.001), confirming that participants were highly motivated to choose between scenarios rather than avoid the decision altogether. However, the small and non-significant difference between ASC_A and ASC_B implies that participants exhibited no systematic bias toward either the first or second scenario across trials.

The estimated coefficients from the multinomial logit model provide insight into the relative importance of each ethical attribute considered in participants’ decision-making. Most attribute levels were statistically significant (*p* < 0.001), indicating that each played a meaningful role in how participants evaluated AV dilemma scenarios (see Table 3). Note that the first level of each attribute is used as a “baseline” value with which the other levels are compared, explaining their value of 0.

**Table 3:** Estimated coefficients per attribute level for critical scenarios.

Attribute	Level	Value	Std Error	*t*-test	*p*-value
Physical harm	Severe injury	0,45	0,09	5,11	<0.01
	Fatality	-0,70	0,08	-8,91	<0.01
Psychological damage	Moderate Distress	-0,99	0,20	−4,98	<0.01
	Severe Trauma	−1,54	0,18	−8,74	<0.01
Moral responsibility	Fully responsible	0,00	0,53	0,00	1
	Partially responsible	−1,40	0,35	−3,97	<0.01
	No responsibility	−1,89	0,37	−5,09	<0.01
	Victim of external factors	−1,47	0,35	−4,23	<0.01
Fair innings	Fully lived life	0,00	0,19	0,00	1
	Close to full life	−0,33	0,20	−1,65	0,10
	Partial life	−1,77	0,63	−2,81	<0.01
	Minimal life lived	−1,30	0,66	−1,96	0,05
Legality of the action	Fully legal	0,00	0,41	0,00	1
	Minor infraction	−1,71	0,38	−4,56	<0.01
	Moderate infraction	−1,24	0,32	−3,81	<0.01
	Severe violation	−1,87	0,26	−7,04	<0.01
Environmental harm	Minimal impact	0,00	0,29	0,00	1
	Low damage	0,49	0,43	1,13	0,26
	Moderate damage	−0,41	0,31	−1,33	0,18
	High impact	−0,60	0,27	−2,25	0,02

In critical scenarios, participants’ preferences were significantly influenced by the levels of each attribute. As expected, physical harm had a strong effect: severe injury was positively weighted (β = 0.455, *p* < 0.001), while fatality carried a significant negative utility (β = −0.697, *p* < 0.001), indicating that participants preferred to minimise loss of life when possible. Psychological damage also had a substantial influence; both moderate distress (β = −0.992, *p* < 0.001) and severe trauma (β = −1.539, *p* < 0.001) reduced the attractiveness of a scenario, with stronger penalties for more extreme distress.

Moral responsibility strongly shaped decision-making: compared to fully responsible individuals (reference), scenarios involving patients with partial or no responsibility, or being victims of external factors, were significantly penalised (βs between −1.40 and −1.90, all *p* < 0.001), suggesting that participants were ethically sensitive to agency and blame. The fair innings (age) effect also appeared meaningful: scenarios involving partial life (β = −1.77, *p* = 0.005) and minimal life lived (β = −1.30, *p* = 0.050) were significantly less preferred than those involving individuals at the end of life, indicating a bias toward preserving younger lives.

**Figure 2:**
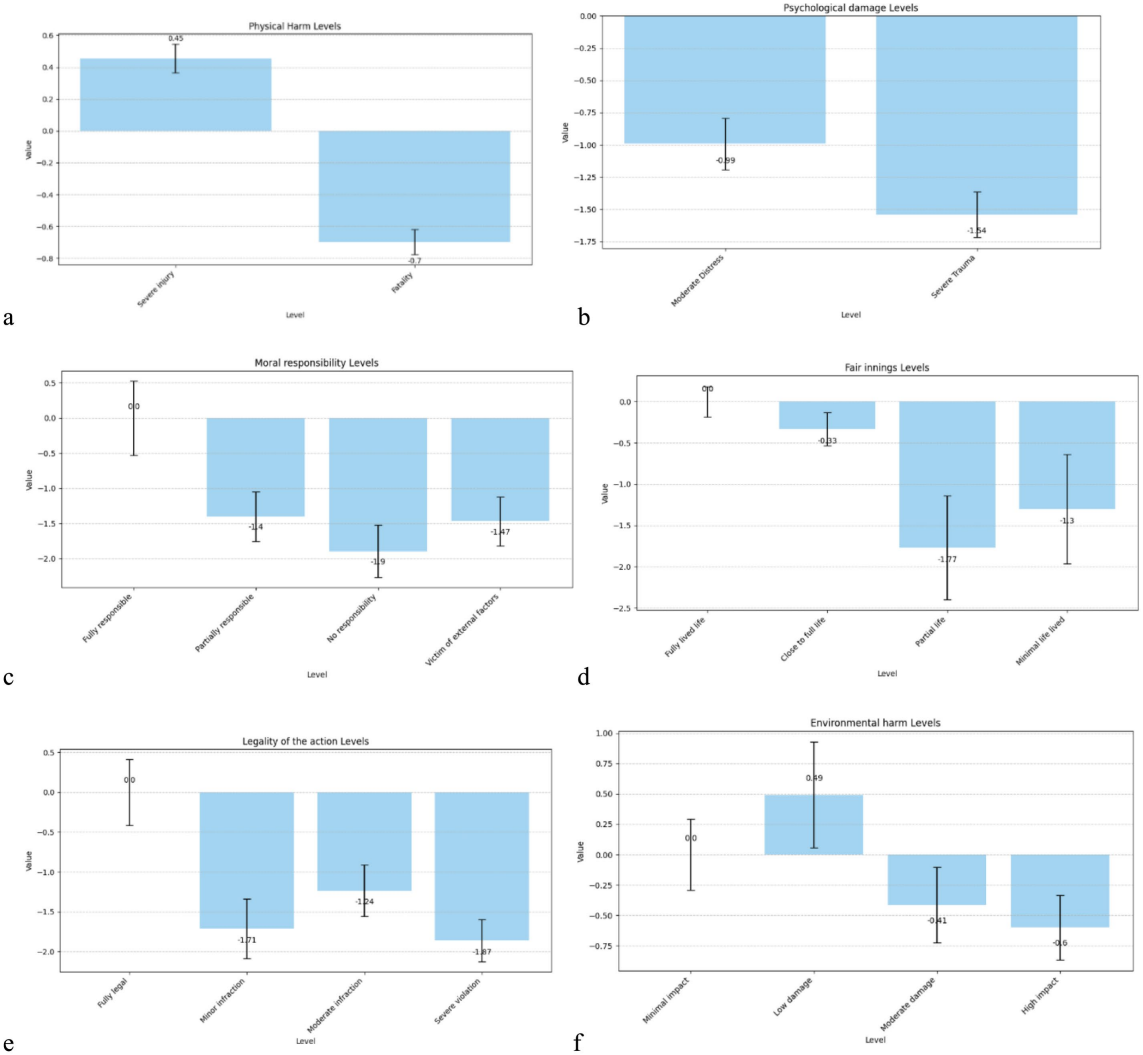
Bar chart of the utility weights of the attribute levels for critical scenarios. **(a)** Physical damage, **(b)** Psychological damage, **(c)** Moral responsibility, **(d)** Fair innings, **(e)** Legality of the Action, **(f)** Environmental harm.

Participants also penalised legal violations by the AV: minor, moderate, and severe infractions all led to significant negative utility (βs between −1.24 and −1.87, *p* < 0.001), with the greatest penalty for severe violations. Environmental harm had weaker effects; only high-impact damage reached significance (β = −0.598, *p* = 0.024), while lower levels were not statistically meaningful (Figure 2).

##### 3.1.1.4 Correlations between attribute levels in critical scenarios

Analysis of the correlations between attribute levels in critical scenarios revealed a complex structure of interdependencies, suggesting that participants’ ethical evaluations were shaped by multidimensional judgments rather than isolated attribute effects. Strong negative correlations were observed between physical harm—severe injury and physical harm—fatality (r = −0.72), as well as between psychological damage—moderate distress and severe trauma (r = −0.95), indicating clear contrasts within each attribute where higher severity levels were distinctly penalized. Moreover, psychological damage—severe trauma showed strong positive correlations with physical harm—severe injury (r = 0.68), and with moral responsibility—fully responsible (r = 0.87), suggesting participants treated high physical and psychological harm as jointly contributing to moral severity, particularly when the victim was deemed responsible.

A similar pattern was seen within the legality dimension, where minor, moderate, and severe violations of legal rules were positively correlated (e.g., r = 0.93 between moderate and severe violations), reflecting a coherent mental grouping of legal breaches. Strong correlations also emerged between fair innings levels, particularly between partial life and minimal life lived (r = 0.82), implying a shared ethical weight for younger lives.

One notable finding was the strong correlation between “moral responsibility—victim of external factors” and “environmental harm—moderate damage” (r = 0.76). While the strength of this relationship is clear, its interpretation is less straightforward. It may reflect a perceived alignment between external causality and broader contextual harms (such as environmental factors), but further investigation would be needed to confirm whether participants genuinely associated these concepts or if this reflects artefacts of scenario design or attribute co-occurrence.

**Figure 3:**
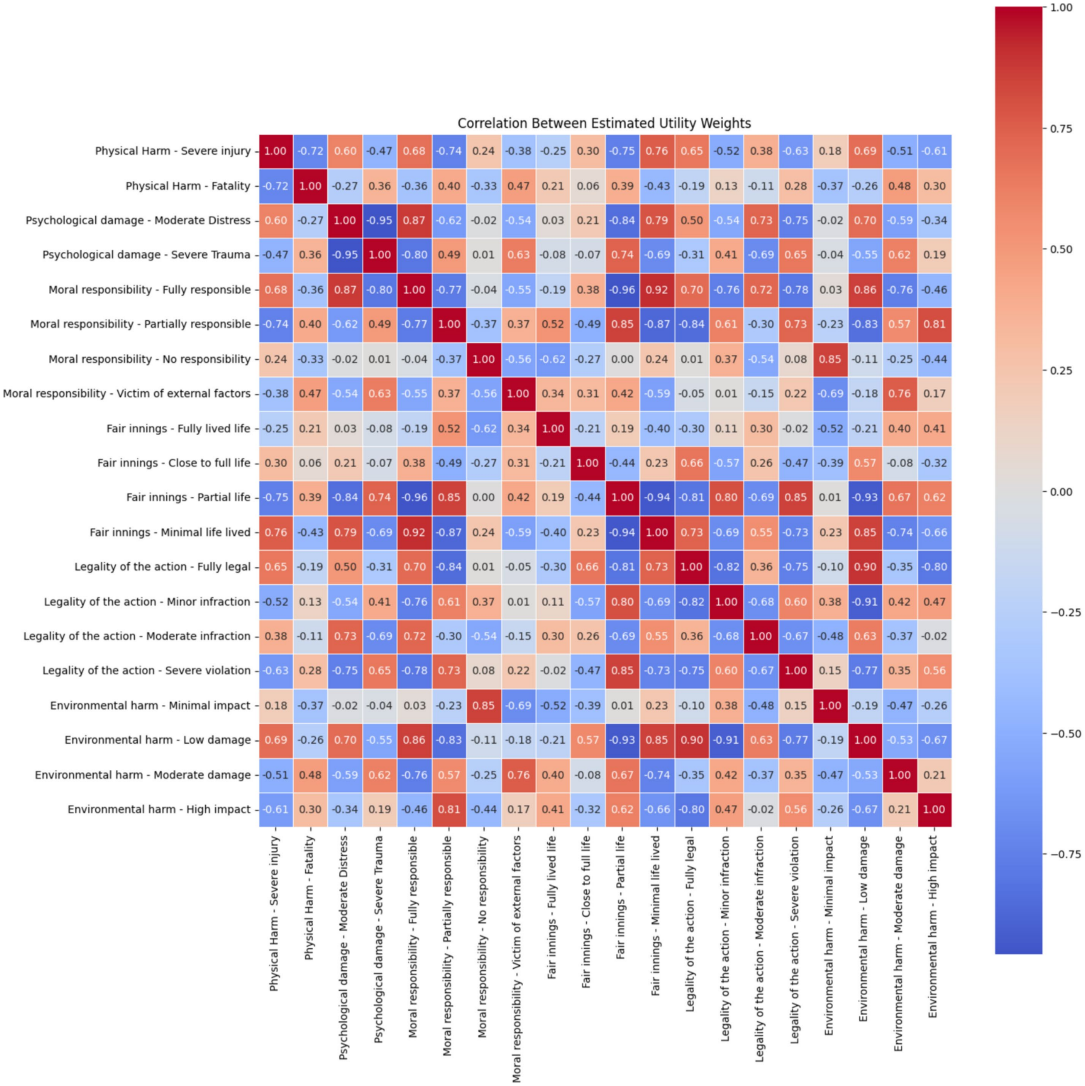
Correlation matrix of individual attribute levels for critical scenarios.

Overall, the correlation structure highlights that while participants distinguish sharply between levels within attributes, they also perceive strong ethical linkages between different moral dimensions, particularly between harm, responsibility, and legal compliance. These associations underscore the importance of modelling moral decision-making with interaction-aware structures that capture how people synthesise multiple sources of harm and blame (Figure 3).

##### 3.1.1.5 Model for non-critical scenarios

The non-critical scenario multinomial logit model was fitted using a dataset of 1,251 valid choice observations and comprised 25 estimated parameters, including utility weights for six ethical attributes and alternative-specific constants. The model showed strong explanatory performance, with a final log-likelihood of −741.48 and a rho-squared value of 0.4605, reflecting a substantial improvement over the null model. Model evaluation criteria supported the robustness of the specification, with an AIC of 1532.96 and a BIC of 1661.25.

The alternative-specific constants revealed a clear tendency among participants to prefer making a choice rather than opting out: both ASC_A (β = 1.68) and ASC_B (β = 1.80) were highly significant (*p* < 0.001), while the no-choice option was strongly rejected (ASC_NC = −3.48, *p* < 0.001). The negligible and non-significant difference between ASC_A and ASC_B suggests no systematic ordering effect between the two presented scenarios.

Most of the attribute levels included in the model significantly influenced participant decisions (*p* < 0.001). The resulting coefficients reflect the relative weight assigned to different moral considerations and are detailed in Table 4.

**Table 4:** Estimated coefficients per attribute level for non-critical scenarios.

Attribute	Level	Value	Std Error	*t*-test	*p*-value
Physical harm	No harm	1,09	0,20	5,59	<0.01
	Minor injury	−0,03	0,21	−0,15	0,88
Psychological damage	No Distress	0,02	0,10	0,18	0,86
	Low Distress	−0,19	0,13	−1,46	0,14
	Moderate Distress	−0,53	0,31	−1,75	0,08
	Severe Trauma	−1,99	0,25	−8,12	<0.01
Moral responsibility	Fully responsible	−0,23	0,16	−1,45	0,15
	Partially responsible	−0,34	0,22	−1,54	0,12
	No responsibility	−0,72	0,17	−4,24	<0.01
	Victim of external factors	−0,91	0,13	−7,11	<0.01
Fair innings	Fully lived life	−0,48	0,15	−3,15	<0.01
	Close to full life	−0,29	0,24	−1,19	0,24
	Partial life	−0,63	0,20	−3,15	<0.01
	Minimal life lived	−0,80	0,13	−5,97	2,39E+06
Legality of the action	Fully legal	0,02	0,12	0,16	0,87
	Minor infraction	−0,58	0,12	−4,73	<0.01
	Moderate infraction	−0,65	0,32	−2,03	0,04
	Severe violation	−0,99	0,27	−3,60	<0.01
Environmental harm	Minimal impact	−0,34	0,11	−3,17	<0.01
	Low damage	−0,32	0,11	−2,89	<0.01
	Moderate damage	−0,54	0,14	−3,84	<0.01
	High impact	−1,00	0,14	−7,24	<0.01

In non-critical scenarios, participants’ choices were influenced by multiple ethical and contextual attributes. The most notable effect was observed for physical harm: participants significantly favoured scenarios involving no harm (β = 1.09, *p* < 0.001) over those involving minor injury, though the latter did not reach significance (β = −0.03, *p* = 0.88), suggesting a strong threshold preference for avoiding all physical harm. Psychological damage also played a substantial role: while low and moderate distress did not individually reach significance, severe trauma had a strong negative effect (β = −1.99, *p* < 0.001), indicating that even in low-physical-harm situations, high psychological impact substantially reduced the attractiveness of a scenario.

Moral responsibility showed a clear gradient: participants were more averse to harming individuals with less responsibility. Scenarios involving no responsibility (β = −0.72, *p* < 0.001) or where the person was a victim of external factors (β = −0.91, *p* < 0.001) were significantly penalised. The fair innings (age-based) effect was also evident: harming those who had fully lived life or had lived a partial or minimal life was increasingly penalised (βs between −0.48 and −0.80, all *p* < 0.01), suggesting sensitivity to life stage, with the greatest concern shown for children and young adults.

**Figure 4:**
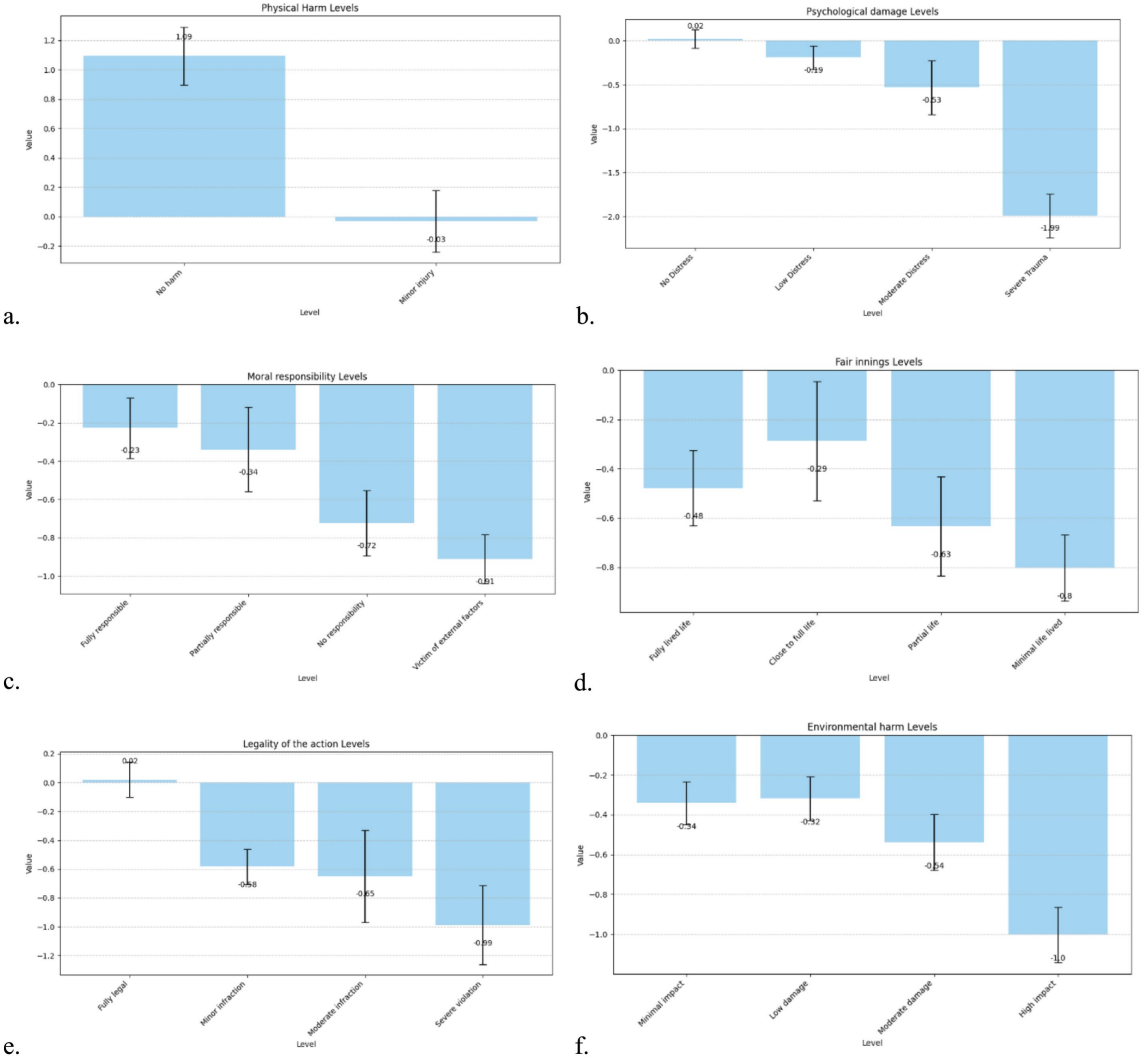
Bar chart of the utility weights of the attribute levels for non-critical scenarios. **(a)** Physical damage, **(b)** Psychological damage, **(c)** Moral responsibility, **(d)** Fair innings, **(e)** Legality of the Action, **(f)** Environmental harm.

The legality of the AV’s action emerged as another critical factor: while minor and moderate infractions carried moderate penalties (β = −0.58 and −0.65, respectively), severe violations had a significantly stronger negative impact (β = −0.99, *p* < 0.001). Participants therefore appear to weigh ethical acceptability against legal compliance even in lower-stakes contexts. Finally, environmental harm was also considered: although all levels had negative weights, only moderate (β = −0.54, *p* < 0.001) and high-impact damage (β = −1.00, *p* < 0.001) reached high significance, indicating that ecological considerations influence preferences but less strongly than direct human harm (Figure 4).

##### 3.1.1.6 Correlations between attribute levels in non-critical scenarios

In non-critical scenarios, the correlation structure between attribute level coefficients revealed nuanced interdependencies, especially among psychological, moral, and environmental dimensions. A strong negative correlation was found between “psychological damage—low distress” and “psychological damage—severe trauma” (r = −0.93), indicating that participants clearly differentiated between mild and severe emotional impacts even in low-risk situations. Similarly, “moral responsibility—partially responsible” was highly negatively correlated with “victim of external factors” (r = −0.76), reflecting consistent ethical penalisation of scenarios in which the harmed party lacked agency.

The legality dimension again showed internal consistency, with strong correlations between minor, moderate, and severe violations (e.g., r = 0.93 between moderate and severe), indicating coherent ethical weighting across levels of rule-breaking. Across other attributes, several moderate to strong correlations were observed—for example, between fair innings—minimal life lived and “psychological damage—severe trauma” (r = 0.77), suggesting that scenarios involving vulnerable individuals (e.g., children) coupled with severe emotional harm carried particular moral weight.

Notably, there is a positive correlation between “psychological damage—moderate distress” and “environmental harm—moderate damage,” with an observed correlation coefficient of r = 0.71. However, it is also possible that individuals interpret the severity of these consequences differently depending on their personal values or experiences, which could influence how they conceptualise the relationship between these forms of harm.

**Figure 5:**
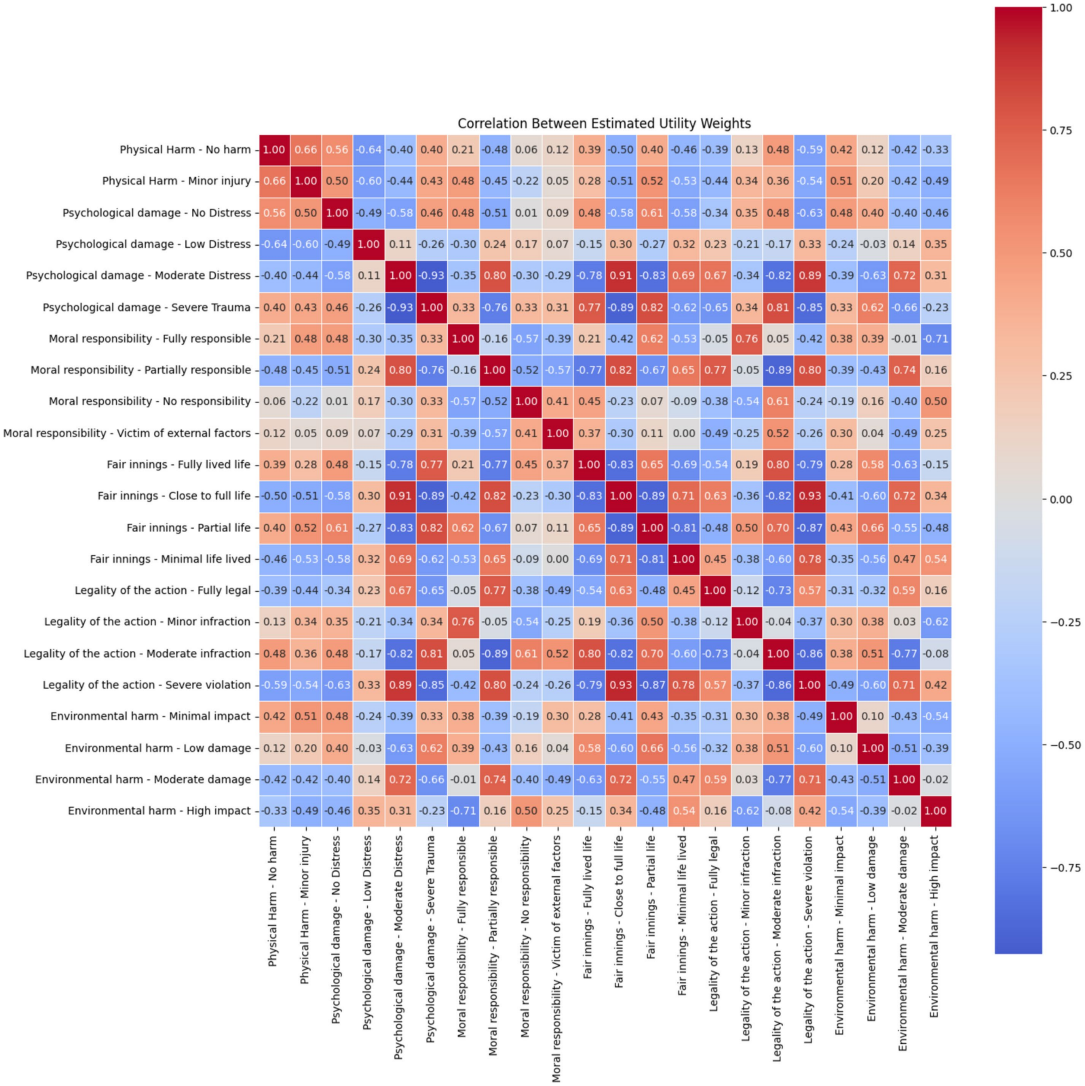
Correlation matrix of individual attribute levels for non-critical scenarios.

Importantly, some attribute levels showed independence: for instance, “environmental harm—low damage” exhibited low correlations with moral or psychological variables, indicating that minor environmental outcomes were evaluated relatively independently of human-centred harms. Overall, the correlation structure reinforces the idea that even in lower-stakes contexts, participants integrated multiple dimensions of harm and responsibility in structured, interpretable ways, highlighting the ethical sensitivity present even in non-lethal decision scenarios (Figure 5).

##### 3.1.1.7 Comparison between critical and non-critical utilities

Comparing the discrete choice model results across critical and non-critical scenarios revealed both consistent ethical patterns and notable contextual shifts in how participants weighted different moral attributes. In both models, participants strongly penalised scenarios involving severe psychological harm. However, the magnitude of the disutility was considerably higher in critical contexts, with severe trauma receiving a large negative coefficient (β = −1.54, *p* < 0.001) in critical scenarios compared to an even larger penalty (β = −1.99, *p* < 0.001) in non-critical ones. This suggests that psychological harm retains salience regardless of overall risk and may even be judged more harshly when physical consequences are low, potentially due to a focus on avoidable suffering.

In terms of moral responsibility, both models showed a clear ethical aversion to harming individuals who were not responsible or victims of external factors, but the effect was stronger in critical scenarios. For instance, the coefficient for ‘no responsibility’ was β = −1.90 in critical contexts (*p* < 0.001), compared to β = −0.72 in non-critical ones (*p* < 0.001), indicating greater moral weight assigned to agency when lives were at stake. Similarly, age-based preferences (“fair innings”) were present in both models, but with differing emphases. In critical scenarios, harming children or young adults was strongly disfavoured (e.g., partial life: β = −1.77), while in non-critical settings, penalties for youth-related harm were more moderate (e.g., minimal life lived: β = −0.80), suggesting that life-stage considerations become more prominent under life-or-death conditions.

Legal violations by the AV were consistently penalised across both contexts. Yet, while severe violations were significantly disfavoured in both models (β = −1.87 critical, β = −0.99 non-critical), legal infractions carried greater relative weight in non-critical settings, potentially because participants used rule-following as a decision anchor in less morally clear-cut cases. Environmental harm was penalised more sharply in non-critical scenarios, with high impact carrying a larger negative utility (β = −1.00, *p* < 0.001), whereas in critical scenarios, environmental damage had a comparatively weaker influence, suggesting that when human lives are at risk, ecological concerns become secondary.

Finally, the alternative-specific constants (ASCs) revealed a stronger tendency to avoid the “no choice” option in both scenarios, but with even stronger preference to act in critical cases (ASC_NC = −4.60) than in non-critical ones (ASC_NC = −3.48). This indicates that participants felt a greater sense of obligation to make a choice when outcomes involved significant physical harm.

Overall, the comparison suggests that while ethical reasoning is consistently structured around harm, responsibility, and legality, the weight of these principles shifts depending on whether the situation is life-threatening or not. These findings reinforce the need for adaptive ethical models in automated systems that respond to contextual stakes, rather than applying uniform moral rules across all scenarios.

##### 3.1.1.8 Predictive performance

To evaluate the determinants of choice behaviour, we estimated separate multinomial logit (MNL) models for the critical and non-critical decision contexts. The models were implemented and validated using five-fold cross-validation to assess their generalization performance.

The initial specification included a large number of attribute-level dummy variables for six attributes, resulting in 23 and 25 estimated coefficients for the critical and non-critical models, respectively. Examination of the correlation matrices revealed substantial multicollinearity, with 13 pairs of parameters in the critical model and 30 pairs in the non-critical model showing absolute correlations above |r| > 0.8. Specifically:

- In the critical model, we dropped or merged parameters showing high correlation or weak statistical significance, such as Moral responsibility—Fully Responsible, Fair innings—Fully lived life, Fair innings—Close to full life, Legality of the action—Moderate infraction, and Environmental harm—Low damage.
- In the non-critical model, parameters including Psychological damage—Low distress, Moral responsibility—Fully responsible, Moral responsibility—Partially responsible, Fair innings—Close to full life, and Legality of the action—Fully legal were removed.

These adjustments reduced the number of free parameters to approximately 14 per model and eliminated the most collinear variable pairs. After simplification, all remaining parameters displayed the expected signs and several achieved strong statistical significance.

The models achieved a mean prediction accuracy of 63.8% (SD = 3.3%) across the five folds when evaluated on choices between the two alternatives (A vs. B), excluding “No Choice” responses. Table 5 presents the detailed results for each fold.

**Table 5:** Cross-validation results by fold.

Fold	Total samples	A/B choices	No choice	Accuracy
1	418	407	11	66.6%
2	418	402	16	63.9%
3	418	405	13	57.5%
4	417	398	19	66.3%
5	417	404	13	64.6%
Mean	417.6	403.2	14.4	63.8%

The optimization procedure converged successfully across all folds, with the Newton trust region algorithm terminating based on relative gradient criteria (≤ 6.1e−06). Most folds required 3–4 iterations for convergence, with optimization times ranging from 7 to 11 s per model. The successful convergence and low number of iterations required suggest stable parameter identification.

The confusion matrices reveal that the models performed reasonably well in discriminating between alternatives A and B:

- Fold 1 demonstrated the highest accuracy (66.6%), with relatively balanced performance across both alternatives
- Fold 3 showed the lowest accuracy (57.5%), indicating greater difficulty in predicting choices in this particular data split
- Across folds, the models showed no strong systematic bias toward predicting either alternative

The “No Choice” option comprised only 3.4% of total responses (averaging 14.4 cases per fold), suggesting that participants generally felt comfortable making a choice between the two scenarios presented. Since the model is constrained to predict either A or B (reflecting a forced-choice decision context), evaluation focused on accuracy for A/B choices only.

#### 3.1.2 Consistency and sensitivity

Participant responses demonstrated a high degree of consistency and thoughtful engagement throughout the experiment. The average switching rate between consecutive choices was 55.82%, indicating that participants adapted their responses based on the specific trade-offs presented, rather than defaulting to one option, highlighting a high sensitivity. Notably, none of the participants consistently chose the same option across all tasks, confirming that no respondent exhibited repetitive or disengaged behaviour.

**Figure 6:**
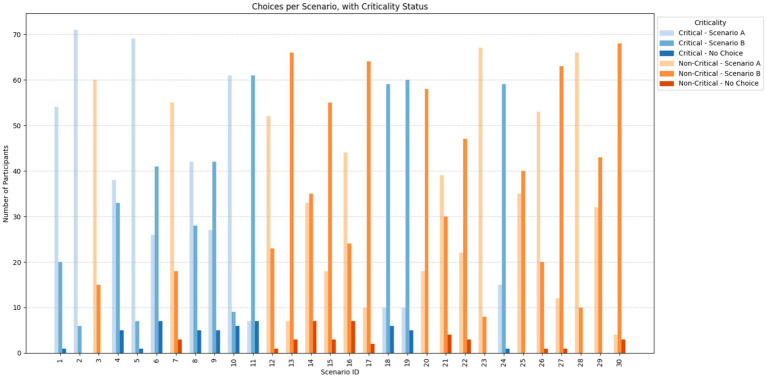
Distribution of choices per question across all participants.

Additionally, model accuracy across time bins remained stable: 70.1% for early tasks, 69.6% for middle tasks, and 69.1% for late tasks. This slight decrease suggests minimal fatigue and does not raise concerns about declining data quality over time. Overall, these indicators confirm that participants responded dynamically to scenario content, and that the experiment design successfully elicited non-random, reliable choices suitable for robust model estimation (Figure 6).

### 3.2 Additional questions

To better understand participants’ experience with the experiment and their views on the ethical attributes used, several additional questions were included after the choice tasks.

First of all, participants were asked, “How confident did you feel about your decisions?.” They had to answer on a 5-point Likert scale, from “Not confident at all” to “Very confident.” Most respondents reported feeling reasonably confident in their decisions. Specifically, 66% said they were somewhat confident, while 8% were very confident. A smaller portion felt neutral (13%), not very confident (10%), or not confident at all (2%). This indicates that, overall, participants felt capable of engaging with the trade-offs presented, though not without some uncertainty.

When asked, “Do you think these attributes completely define AV decision-making? If not, what should be added?,” only 19% answered yes. 48% responded maybe, and 33% said no. This suggests that while the attributes captured key considerations, many participants felt the framework might benefit from additional or alternative dimensions. However, most of the comments suggested either attributes already taken into account but on a larger scale (physical harm to the occupants of the AV) or attributes that had been removed from the experiment for simplicity (social status, damage to the AV). Many participants also requested more context, such as weather conditions or uncertainty, however these parameters would have been taken into account by the AV in the computation of the estimation of the attributes.

To get a broader understanding of the decision-making process of participants, they were then asked to select which attributes should always be taken into account. Among the six core attributes, Physical Harm was overwhelmingly viewed as essential, selected by 100% of participants. Other commonly endorsed attributes included Psychological Damage (21%), Moral Responsibility (15%), and Legality (13%). Fewer participants selected Fair Innings (10%) and Environmental Harm (9%) as always relevant. This distribution highlights a prioritisation of direct human impact in ethical AV decision-making.

When asked which attributes should never be considered, 30% of respondents believed that none of them should be excluded outright. Environmental Harm (23%) and Legality (21%) were the most frequently selected attributes to never be considered, followed by Fair Innings (12%) and Moral Responsibility (8%). These results suggest some scepticism about the relevance of less immediate or more abstract dimensions in AV ethics.

When asked to explain their decision-making (“Please describe how you made your decisions in the scenarios presented. What factors or attributes did you prioritise? Did any particular attribute play a major role in your choices?”), participants most frequently identified Physical Harm as the most important attribute guiding their choices. This was followed by Psychological Harm, Moral Responsibility, and Fair Innings. In contrast, Legality and Environmental Harm were less commonly cited as influential factors in participants’ reasoning.

Several participants elaborated on how different attributes interacted in shaping their decisions. For instance, Moral Responsibility was often deemed less critical when the affected individual was a child, suggesting that age modulated the perceived moral obligations. Others described complex trade-offs, such as prioritizing the death of an older adult (80 + years old) over subjecting a child to a lifetime of PTSD, indicating a nuanced consideration of both age and long-term psychological harm.

When reflecting on their confidence in decision-making, participants frequently expressed uncertainty, particularly around the attributes of Moral Responsibility and Fair Innings. Many questioned whether it is justifiable for individuals deemed more morally responsible to endure more serious injuries, indicating discomfort with the ethical implications of such trade-offs. Similarly, the concept of Fair Innings elicited mixed feelings, with participants feeling unsure how to consistently apply age-based value judgments. Some explicitly noted their lack of confidence in the consistency of their own choices, admitting they might respond differently if presented with the same scenario again. This highlights the inherent complexity and subjectivity involved in balancing competing ethical considerations.

### 3.3 Validation of human rating methodology

Individual ethical preferences naturally vary across participants, particularly in complex moral contexts such as AV decision-making. To ensure that our dataset captured consistent and interpretable patterns rather than random variation, we conducted multiple quality and reliability checks. First, dominance and attention checks were embedded in the questionnaire to identify inattentive or inconsistent respondents, whose data were subsequently excluded. Second, we evaluated behavioural consistency through the proportion of logically consistent responses and switching rates across choice sets. Third, to evaluate the reliability of participants’ moral judgments, we computed Krippendorff’s alpha (α), which measures inter-rater agreement while accommodating missing data and varying numbers of ratings per item (Krippendorff, 2019). This approach was chosen because participants were randomly assigned to evaluate 24 out of 30 scenarios, resulting in an incomplete design unsuitable for traditional measures such as Cohen’s or Fleiss’ *κ*. The resulting coefficient, α = 0.29, indicates *fair to moderate agreement* among participants (Landis and Koch, 1977).

## 4 Discussion

### 4.1 Experimental results

The findings reveal a number of interesting tensions in participants’ moral reasoning. Notably, while the concept of Fair Innings—the idea that younger individuals should be prioritised over older ones—was frequently cited as ethically problematic (and even referenced as legally prohibited, for example in German law (Luetge, 2017), it nonetheless remained one of the most commonly selected attributes in decision-making. This suggests a gap between participants’ stated principles and their practical choices, reflecting the complexity of applying abstract ethical norms to concrete scenarios.

Similarly, Legality was generally seen as less important in guiding decisions, a result that may appear counterintuitive given the regulatory and institutional role of legal frameworks in moral dilemmas. This downplaying of legality may point to participants prioritising personal or moral intuitions over institutional norms when forced to make difficult trade-offs.

Several participants also reported feeling uncertain or lacking confidence in their decisions, particularly due to limited contextual understanding of the scenarios. Comments such as “I do not really know the situation all that well” and “I’d be more confident if I could see the situation” suggest that the mode of presentation plays a crucial role in shaping decision-making. This aligns with prior research by Gros et al. [ref lit review & exp2], which emphasizes how richer, more immersive media—such as 3D animations or VR simulations—can enhance understanding and engagement in complex ethical scenarios. Enhancing the realism and clarity of these scenarios may therefore improve both the quality of participants’ judgments and their confidence in making them.

Overall, these findings highlight not only the difficulty of ethical decision-making in abstract contexts but also the importance of how information is presented. Future work could explore how different presentation modes affect moral intuitions and consistency, potentially informing the design of decision-support tools in high-stakes ethical domains.

### 4.2 Model evaluation

The model performed reasonably across our key evaluation criteria. Consistency was high: participants rarely gave random answers, and dominance and attention check results confirmed logical engagement. The switching rate was within a healthy range, and no participants consistently selected the same scenario, indicating active trade-off evaluation. The model also demonstrated good sensitivity, with clear variation in preferences across different attribute levels. Confidence ratings were generally positive, suggesting that participants understood the tasks and were comfortable with the ethical trade-offs.

The mean accuracy of 63.8% substantially exceeds the 50% baseline expected from random guessing in a binary choice task, demonstrating that the models successfully captured systematic patterns in participant decision-making. The modest standard deviation (3.3%) across folds indicates stable performance, though Fold 3’s lower accuracy suggests some sensitivity to specific data compositions.

The separate modelling of critical versus non-critical scenarios allowed for potential differences in attribute weights between high-stakes and low-stakes ethical decisions. Model convergence with relatively few iterations across all folds supports the validity of this modelling approach, indicating that the harm-based segmentation strategy was appropriate for this dataset.

It is important to note that these models were deliberately specified as parsimonious multinomial logit models to ensure interpretability and avoid overfitting. We removed highly correlated parameters during model development to maintain numerical stability and avoid multicollinearity issues. As such, the 63.8% accuracy should be viewed as optimistic rather than limiting, particularly given the complexity of ethical decision-making and the simplified model structure.

The consistent performance across folds (ranging from 57.5 to 66.6%) demonstrates that the simplified model captures robust, generalizable patterns in choice behaviour rather than fold-specific noise. This stability is particularly valuable for understanding the systematic drivers of ethical preferences in our population.

While the 63.8% accuracy demonstrates above-chance prediction with a deliberately simplified model, several avenues exist for future research. The removal of highly correlated parameters, while necessary for model stability, may have constrained the model’s ability to distinguish between subtle attribute level differences. In addition, the effect of interactions between attributes has not been analysed and could add some complexity to the model, further improving accuracy.

Future work could explore: (1) mixed logit models to capture preference heterogeneity across individuals, allowing for random coefficients that vary in the population; (2) interaction effects between attributes to capture non-additive decision-making patterns (e.g., harm type interacting with probability); (3) alternative model specifications such as nested logit if choice correlations exist; and (4) incorporation of individual-level covariates (demographics, moral foundations) to explain systematic preference variation.

However, any movement toward model complexity must be balanced against the risk of overfitting, particularly given the relatively modest sample sizes when data are split by harm level and cross-validation fold. The current parsimonious approach provides a robust baseline for understanding ethical decision-making patterns and demonstrates that systematic, generalizable preferences can be captured even with simplified model structures. As such, the constraints detailed here are intentional: they provide a necessary starting point for operationalising ethical preferences into a computational framework. We see this model not as a complete ethical engine for AVs, but as a first step in building more refined, responsive, and human-centred decision systems.

### 4.3 Operationalising the critical/non-critical distinction

This study introduces a critical distinction in ethical AV decision-making: the separation of critical and non-critical scenarios, operationalised via a two-stage decision model. This distinction, grounded in empirical behaviour, allowed us to model shifts in ethical prioritisation based on the perceived severity of harm, an approach that aligns with how humans intuitively allocate moral weight in high- versus low-stakes situations.

The results from our discrete choice models validate this dual-structure approach. While core ethical concerns—such as harm, responsibility, age, and legality—remained influential across both critical and non-critical contexts, their relative weight shifted significantly depending on scenario type. For example, although severe psychological harm was always penalised, it received a stronger disutility in non-critical scenarios (β = −1.99) than in critical ones (β = −1.54), suggesting that participants paid more attention to avoidable suffering when the physical risks were lower. Similarly, moral responsibility carried greater weight in critical scenarios (β = −1.90 vs. –0.72), reinforcing the idea that judgments of agency and fairness become even more morally salient when lives are at stake.

In practice, however, the first challenge lies in the real-time classification of whether a given situation is critical or non-critical. This would require AVs to make fast and reliable assessments of risk severity, likely through a combination of sensor data, predictive modelling, and pre-defined thresholds for outcomes such as probability and magnitude of physical harm. For example, scenarios with high-speed trajectories, vulnerable road users, or imminent collisions could be flagged as “critical,” triggering a simplified decision logic that prioritises harm minimisation. Conversely, ambiguous or low-stakes situations—e.g., choosing between minor delays or increased fuel consumption—could be routed through a more complex, multi-attribute ethical evaluation. Importantly, these classifications are not necessarily static: risk minimisation strategies (e.g., braking, steering adjustments) may successfully reduce the likelihood or severity of harm, effectively de-escalating a critical scenario into a non-critical one. This dynamic progression implies that both levels of ethical evaluation could apply at different time points within the same unfolding event, highlighting the need for flexible, real-time ethical goal functions that can transition between modes as the situation evolves.

Furthermore, this framework raises important ethical and technical questions. The legitimacy of treating scenarios differently must be scrutinised: Should ethical priorities shift with risk severity, or should the same principles apply universally? Technically, determining thresholds between critical and non-critical scenarios introduces potential grey zones where misclassification could result in suboptimal or ethically inconsistent outcomes. These risks highlight the need for transparent classification criteria and robust safeguards.

The critical/non-critical distinction offers a promising path forward for scalable and context-aware ethical AV behaviour. However, it must be operationalised with caution, combining robust risk assessment with normative safeguards to ensure consistency, transparency, and public trust.

### 4.4 Inter-rater agreement

An important consideration when aggregating individual preferences into a collective ethical goal function is the degree of consensus among participants. We assessed inter-rater reliability using Krippendorff’s alpha (α), which measures the agreement among participants (raters) when evaluating the same ethical scenarios (items). Our analysis yielded α = 0.29, indicating fair to moderate agreement across the 30 experimental scenarios. While this value exceeds the threshold for random agreement (α = 0), it falls below conventional benchmarks for high reliability [α > 0.67 for tentative conclusions; α > 0.80 for definitive conclusions (Krippendorff, 2019)].

This modest inter-rater reliability reflects a fundamental characteristic of ethical decision-making: individuals hold legitimately different moral values, priorities, and reasoning frameworks when evaluating ethical trade-offs. The observed preference heterogeneity is not necessarily indicative of measurement error or data quality issues—indeed, our data quality controls (attention checks, low “No Choice” rates of 3.4%, and systematic patterns evidenced by 63.8% predictive accuracy) suggest participants were engaged and expressing genuine preferences. Rather, the relatively low agreement highlights that ethical preferences are inherently diverse, even within relatively homogeneous populations.

Our aggregated goal function represents the central tendency of preferences within our sample (university students) rather than universal ethical principles. This is appropriate for a feasibility study demonstrating that ethical goal functions can be empirically derived and operationalized for AV alignment. However, it limits generalizability beyond similar populations. However, while this sample is often assumed to be relatively homogeneous in terms of demographic background, it is not clear whether such groups are in fact homogeneous in their moral values. Likewise, working with a more formally heterogeneous population does not necessarily imply either higher or lower inter-rater reliability. Preference heterogeneity therefore remains an important empirical question for future research and a key challenge for AV ethics applications.

Notably, inter-rater reliability is rarely reported in empirical ethics research on AV decision-making or moral machine-type experiments. To our knowledge, no published studies in this domain report Krippendorff’s alpha or similar agreement metrics, making direct comparison challenging. However, variation in moral judgments is not a flaw unique to this study, but an inherent and well-recognized feature of ethical decision-making. Just as democratic political systems operate despite (and because of) persistent value pluralism, AV ethics must also be designed to handle areas of both agreement and disagreement. Our contribution here is therefore not to resolve moral disagreement, but to make patterns of consensus and divergence transparent, so that they can be explicitly considered in the design of alignment processes.

Our findings suggest several important avenues for future research. First, preference segmentation approaches (latent class models, cluster analysis) could identify subgroups with higher internal agreement, allowing for multiple goal functions representing different ethical perspectives. Second, mixed logit models could characterize the full distribution of preferences rather than reducing them to population averages. Third, researchers should investigate whether more diverse samples yield lower agreement and whether certain decision contexts (e.g., extreme vs. ambiguous scenarios) generate more consensus. Finally, the field needs to grapple with normative questions about whose preferences should guide automated ethical decisions when consensus is lacking.

### 4.5 Next steps

Having developed an initial ethical goal function for AV decision-making through a structured choice modelling approach, the next crucial phase involves evaluating whether the resulting decisions are considered acceptable and justifiable by broader society. To enable this, we will implement the Socio-Technological Feedback (SOTEF) Loop—a dynamic, iterative framework that integrates public input, legal oversight, and design refinement to align AV behaviour with societal values (Aliman et al., 2019; Heijnen et al., 2024).

The SOTEF Loop comprises four interconnected feedback loops—Governance, Design, Development, and Operation—each contributing to the continual assessment and evolution of AV ethical systems. The Design Loop is where societal input, like the ethical preferences modelled in this study, is operationalised into concrete behavioural rules for AVs. The Governance Loop ensures that these rules respect regulatory and moral boundaries while remaining flexible to adapt to changing expectations. The Development implements the design specifications into a functioning human-AI system through technological development, validation, and verification, ensuring compliance with ethical and legal standards. Finally, the Operation Loop manages the real-world interaction between humans and AI, involving configuration for specific contexts and adaptation during operation. This loop also provides feedback to refine the other loops.

The next step is to test this ethical goal function in realistic, scenario-based simulations, where AV decisions based on the current model will be presented to participants. Participants will assess whether these decisions align with their ethical expectations. Importantly, these simulations should not only display the chosen outcomes but also transparently communicate the underlying ethical reasoning, such as which attributes were prioritised and why certain trade-offs were made. By exposing participants to both the decision and the rationale behind it, the evaluation moves beyond mere consequential judgement and enables a deeper assessment of value alignment. This feedback will inform whether and how the model needs to evolve.

This process is not only about validating the current model but also about complexifying and refining it. For instance, if participants consistently disagree with decisions involving moral responsibility or legality, this may indicate the need for non-linear relationships, interactions between attributes, or even entirely new moral dimensions not currently included. The SOTEF Loop facilitates this iterative improvement, allowing the model to grow in sophistication over time with societal input, incorporating context-dependence, individual variability, and moral pluralism.

It is important to note that our data reflects the preferences of a Dutch population. Ethical intuitions are shaped not only by individual psychology but also by national legal systems, cultural traditions, and social norms. As such, the relative importance of attributes such as legality, fairness, or harm minimisation may differ across jurisdictions. We therefore do not see this framework as prescribing a single universal model. Instead, the methodology is designed to be replicable: similar discrete choice experiments can be conducted in other cultural and legal contexts to derive context-specific ethical weights. Through the SOTEF Loop, these national or regional models can be integrated, ensuring that automated vehicles remain aligned with local societal expectations while still operating within a shared methodological framework. This flexibility makes the approach both scalable and adaptable across jurisdictions.

Ultimately, this feedback-driven approach ensures that AV decision-making is not frozen in time or isolated from society. Instead, it becomes a living system, continuously shaped by ethical deliberation, empirical data, and human values. In doing so, we move closer to AVs that are not only safe and efficient but also ethically transparent and socially responsive.

Overall, this work also serves a broader proof-of-concept purpose: to demonstrate that it is possible to formally represent moral judgment in a mathematically tractable way. By building and testing ethical goal functions that reflect real human preferences, and iteratively improving them through structured feedback, this work proposes a concrete framework for capturing moral nuance within algorithmic systems, showing that computational ethics need not be a black box or an afterthought, but a deliberate and adaptive component of AV design.

## 5 Conclusion

This study presents a comprehensive framework for building an ethical goal function for automated vehicles (AVs), grounded in empirical data and designed for iterative societal feedback. The process starts with the selection of moral attributes using the Augmented Utilitarianism (AU) framework, supported by insights from moral psychology, biomedical ethics, and neuroscience. These attributes, including physical harm, psychological damage, legality, moral responsibility, fair innings, and environmental harm, were identified as key dimensions in AV ethical decision-making.

To quantify the relative importance of these attributes, we designed a discrete choice experiment, carefully constructed using efficient experimental design methods. To address the dominance of certain attributes (e.g., physical harm), we implemented a two-tiered decision model that distinguishes between “critical” and “non-critical” scenarios. This allowed us to disentangle and meaningfully assess the contribution of less dominant attributes in ethically charged situations.

Data was collected through a robust questionnaire involving 89 participants, with careful attention to consistency, attention checks, and cognitive load. The resulting multinomial logit model revealed clear and statistically significant utility weights for all six tested attributes. Notably, physical harm and psychological damage were rated as most influential, while attributes such as legality and environmental harm were still impactful but less dominant. The model achieved strong goodness-of-fit scores, high participant consistency, and strong predictive accuracy, indicating that the defined attributes capture real ethical reasoning in complex AV scenarios.

This work contributes to the AV ethics literature in three key ways. First, it shifts from theoretical ethics to an actual applied, data-driven, and participatory approach. Rather than relying solely on expert opinion or philosophical doctrine, we directly involved human participants in shaping the decision-making rules for AVs. Second, it provides a concrete empirical methodology for translating abstract ethical principles into a formalized, testable computational model. Whereas prior approaches have largely focused on normative theorizing or survey-based moral judgments, this study demonstrates the feasibility of constructing and validating an Ethical Goal Function (EGF) that captures societal moral preferences through Discrete Choice Modelling. Finally, it introduces the Socio-Technological Feedback (SOTEF) Loop as a mechanism for continuous ethical alignment of AV systems with evolving societal values. Together, these contributions bridge the persistent gap between ethical theory and algorithmic implementation, offering both a proof of concept and a replicable framework for operationalizing moral decision-making in intelligent systems.

## Data Availability

The datasets presented in this study can be found in online repositories. The names of the repository/repositories and accession number(s) can be found at: doi: 10.24416/UU01-5CTCZN.
